# Genomic Characterization of DArT Markers Based on High-Density Linkage Analysis and Physical Mapping to the *Eucalyptus* Genome

**DOI:** 10.1371/journal.pone.0044684

**Published:** 2012-09-11

**Authors:** César D. Petroli, Carolina P. Sansaloni, Jason Carling, Dorothy A. Steane, René E. Vaillancourt, Alexander A. Myburg, Orzenil Bonfim da Silva, Georgios Joannis Pappas, Andrzej Kilian, Dario Grattapaglia

**Affiliations:** 1 Plant Genetics Laboratory, EMBRAPA Genetic Resources and Biotechnology, Brasilia, Brazil; 2 Department of Cell Biology, Universidade de Brasilia, Brasília – DF, Brazil; 3 Diversity Arrays Technology Pty Ltd., Yarralumla, Australia; 4 School of Plant Science and CRC for Forestry, University of Tasmania, Hobart, Tasmania, Australia; 5 Faculty of Science, Health, Education and Engineering - ML12, University of the Sunshine Coast, Maroochydore DC, Queensland, Australia; 6 Department of Genetics, Forestry and Agricultural Biotechnology Institute (FABI), University of Pretoria, Pretoria, South Africa; 7 Genomic Sciences Program - Universidade Católica de Brasília, Brasília – DF, Brazil; Nanjing Forestry University, China

## Abstract

Diversity Arrays Technology (DArT) provides a robust, high throughput, cost-effective method to query thousands of sequence polymorphisms in a single assay. Despite the extensive use of this genotyping platform for numerous plant species, little is known regarding the sequence attributes and genome-wide distribution of DArT markers. We investigated the genomic properties of the 7,680 DArT marker probes of a *Eucalyptus* array, by sequencing them, constructing a high density linkage map and carrying out detailed physical mapping analyses to the *Eucalyptus grandis* reference genome. A consensus linkage map with 2,274 DArT markers anchored to 210 microsatellites and a framework map, with improved support for ordering, displayed extensive collinearity with the genome sequence. Only 1.4 Mbp of the 75 Mbp of still unplaced scaffold sequence was captured by 45 linkage mapped but physically unaligned markers to the 11 main *Eucalyptus* pseudochromosomes, providing compelling evidence for the quality and completeness of the current *Eucalyptus* genome assembly. A highly significant correspondence was found between the locations of DArT markers and predicted gene models, while most of the 89 DArT probes unaligned to the genome correspond to sequences likely absent in *E. grandis*, consistent with the pan-genomic feature of this multi-*Eucalyptus* species DArT array. These comprehensive linkage-to-physical mapping analyses provide novel data regarding the genomic attributes of DArT markers in plant genomes in general and for *Eucalyptus* in particular. DArT markers preferentially target the gene space and display a largely homogeneous distribution across the genome, thereby providing superb coverage for mapping and genome-wide applications in breeding and diversity studies. Data reported on these ubiquitous properties of DArT markers will be particularly valuable to researchers working on less-studied crop species who already count on DArT genotyping arrays but for which no reference genome is yet available to allow such detailed characterization.

## Introduction

DNA marker technologies for high throughput genome-wide genotyping at affordable costs have become indispensable in the plant geneticist’s toolbox. A large array of methods to detect DNA sequence polymorphisms among individual plants have been developed and used widely in the last twenty five years. Although DNA based hybridization inaugurated this journey with RFLP markers [1], PCR-based methods [2], [3] were responsible for removing the barrier to entry in plant genomic analysis for a large number of species, including orphan crops and many forest trees. Most PCR-based molecular marker methods, however, are low throughput and mobility-based, and therefore too time consuming and costly for applications that require genotyping thousands of samples for thousands of markers within modest budgets. Although large SNP arrays have been developed for an increasing number of plant species [4], they still remain largely limited to the major crops and their costs per sample are unaffordable for most plant breeding and germplasm conservation programs.

Diversity Arrays Technology (DArT) was described over a decade ago [5] and has experienced increasing interest in recent years as a robust, high throughput, cost-effective genome-wide method to assay thousands of presence/absence polymorphisms in a single assay. Although proprietary, this technique is licensed freely under an open-source model [6], a condition that has stimulated the development of genotyping arrays for more than 60 organisms including many less privileged crops [7], [8], [9], [10], [11], [12], [13], [14], [15], [16], [17], [18], [19], [20], [21]. DArT involves the isolation and cloning of a random set of DNA fragments from a complexity-reduced DNA sample assembled by pooling several germplasm accessions so that a representative collection of variable genomic sequences of one or more target species is captured. Several thousand of these DNA clones are arrayed on a glass slide and interrogated with a similarly complexity-reduced, PCR-amplified genomic sample. Being a DNA-DNA hybridization-based method using relatively long probes (∼300–500 bp), DArT provides high and consistent signal to noise ratio even across related taxa [22].

In spite of the extensive use of this genotyping platform for many plant species, very little is known regarding the genomic attributes of the DArT array probes that generate the several thousand markers genotyped. With the exception of a study in oats [23], and recent small scale surveys of a few hundred DArT probe sequences in tomato [16] and apple [24], to the best of our knowledge complete DArT arrays have not yet been examined at the sequence level for redundancy, genome coverage and gene content. Additionally, no information is available about the distribution of DArT markers across a genome, mainly because no reference assembly has yet been available for most species where this technology has been used.

A high density DArT genotyping microarray with 7,680 selected probes from a wide representation of 64 *Eucalyptus* species was recently developed [25]. The genus *Eucalyptus* includes over 700 species some of which are the most widely planted hardwood trees worldwide [26]. A particularly outstanding feature of this hybridization-based genotyping tool has been its genus-wide transferability across species, an attribute hardly offered by microsatellites or SNPs [27]. DArT has provided a standardized high-throughput genotyping platform, whereby thousands of markers can be readily assayed in parallel for thousands of samples across *Eucalyptus* species. This DArT array has demonstrated excellent performance for complex phylogenetic and diversity analyses [22], genomic selection [28] and linkage mapping [25], [29], [30]. A detailed understanding regarding the sequence content and genome-wide distribution of the DNA probes that compose this DArT array should greatly expand its value for comparative QTL mapping studies, to navigate from linkage maps to the reference sequence in positional cloning projects and to extract additional genomic information from uniquely informative markers identified in phylogenetic, population genetics and Genomic Selection studies.

Genetic linkage maps have been pivotal tools for examining the inheritance of qualitative and quantitative traits, for comparative mapping, whole genome assembly and for molecular breeding applications, including germplasm analyses, marker-assisted selection and map-based cloning [31]. Linkage maps for species of *Eucalyptus* have been reported for several pedigrees, both intra- and inter-specific, using different molecular marker technologies [32], [33]. Extensive linkage mapping data of anonymous markers has been accumulated with dominant RAPD and AFLP technologies [34], [35], [36], [37], [38], [39], while RFLPs [40], [41] and a recent Single Feature Polymorphisms (SFP) genotyping array [42] have allowed positioning hundreds of genes on existing maps. In spite of all these advances, these marker technologies have not provided a widely applicable tool that can be used to link genotypes to phenotypes in a broader and more sustainable way that includes comparative mapping, gene discovery and genome assisted breeding. Recently, DArT markers have provided the coverage and high-density mapping required to move in that direction [29], [30], although they are still lacking a deeper characterization of their genomic content.

In this study we investigated the genomic properties of the 7,680 DArT marker probes that populate the *Eucalyptus* array by sequencing them, constructing a high density linkage map and carrying out detailed physical mapping analyses using the recently released *Eucalyptus grandis* reference genome sequence (www.phytozome.net). We were specifically interested in: (1) verifying DArT marker performance for linkage mapping, i.e. level of polymorphism, locus ordering and genome coverage; (2) characterizing the sequence composition of the DArT array probes regarding sequence redundancy and gene content; (3) assessing the physical distribution of the DArT marker probes in terms of overall genome coverage and distance from predicted gene models; (4) aligning the linkage map to the corresponding pseudochromosome scaffolds to assess the consistency of physical- *versus* recombination-based locus ordering; and (5) providing pseudochromosome level and genome wide estimates of the relationship between physical and recombination distances.

## Materials and Methods

### Plant Material

A mapping population of 177 F_1_ individuals was derived from an inter-specific cross between two highly heterozygous elite trees, *E. grandis* (clone G38) and *E. urophylla* (clone U15). Both species are widely planted in the tropics and belong to the same subgenus, *Symphyomyrtus*. This mapping pedigree, named GxU-IP was selected as a reference pedigree for mapping purposes in the Genolyptus project [43], immortalized by mini-cutting propagation and planted in a replicated trial in five locations in July 2003 in randomized blocks with single tree plot with five replicates per location. Genomic DNA was extracted from both parents and all F_1_ individuals using 150 mg of leaf tissue stored at –20°C as described previously [34]; the resulting DNA samples were of consistent quality and suitable for DArT and microsatellite genotyping.

### Microsatellite Genotyping

Screening of 300 EMBRA microsatellite markers [44], [45], [46] for polymorphism between the two parents with the additional analysis of six F1 progeny individuals to verify segregation, resulted in the selection of 222 informative microsatellites**.** Microsatellite genotyping was carried out in multiplexed systems with multi-fluorescence detection in an ABI 3100XL as described earlier [45], [47].

### DArT Genotyping

A detailed account of the methods used to prepare the high density *Eucalyptus* DArT array was reported earlier [25]. Briefly, 18 reduced representation *Pst*I/*Taq*I genomic libraries involving a total of 64 different *Eucalyptus* species were built and 23,808 DNA probes were screened in a panel of 96 individuals. A set of 7,680 probes that revealed robust polymorphisms was selected and used to construct the operational DArT genotyping array. This procedure optimized (1) sampling of a large collection of sequence variants to increase recovery of polymorphic clones; and (2) inter-specific transferability of the scored markers. Genomic representations of the two parents and 177 F_1_ individuals of the mapping population were generated with the same complexity reduction method used to prepare the library to generate ‘targets’ for hybridizing to the arrays. After hybridization, microarray slides were washed and scanned using a TECAN LS300 confocal laser microarray scanner at a resolution of 20 µm per pixel with sequential acquisition of 3 images for each microarray slide. The signal from the FAM-labeled vector polylinker provided a reference value for quantity of amplified DNA fragment present in each ‘spot’ of the microarray. The resulting images were analyzed using *DArTSoft* version 7.44, a program created by *Diversity Arrays Technology Pty. Ltd.* for microarray image data extraction, polymorphism detection, and marker scoring. A relative hybridization intensity value was then calculated for all accepted spots as log [Cy-3 signal/FAM signal] for the targets labelled with Cy-3, and log [Cy-5 signal/FAM signal] for targets labelled with Cy-5. *DArTSoft* then compared the relative intensity values obtained for each clone across all slides/targets to detect the presence of clusters of higher and lower values corresponding to marker scores of ‘1’ and ‘0’ respectively. Targets with relative intensity values that could not be assigned to either of the clusters were recorded as missing data. Standard methods of marker discovery were deployed using a combination of parameters automatically extracted from the array data using DArTsoft. The following parameters were used: (1) reproducibility ≥95% as measured by the concordance of the genotype call between technical replicates (replicated targets processed for a minimum of 30% of the DNA samples genotyped); (2) marker quality Q ≥65, which measures between-cluster variance as a percentage of total variance in fluorescent signal distribution among tested samples; and (3) marker call rate ≥75% (percentage of targets able to be scored as ‘0’ or ‘1’).

### Genetic Map Construction

A single integrated genetic linkage map was constructed using both the co-dominant microsatellite data and the dominant DArT marker data using JoinMap v3.0 [48]. Microsatellite markers segregated either from each single parent in a 1∶1 ratio, from both parents in a 1∶2:1 ratio following a phase-unknown F2 configuration with both parents equally heterozygous for the same genotype, or in a fully informative 1∶1:1∶1 ratio with three or four different alleles segregating from the two parents. Dominant DArT markers, on the other hand, segregated either in a 1∶1 pseudo-testcross configuration from each single parent or in a 3∶1 ratio when both parents where heterozygous. For both the microsatellite and DArT data, markers that showed ≥75% call rate and fitted one of the expected segregation ratios at α≥0.01 were used for linkage analysis. The grouping and ordering of the markers were established initially by applying the maximum likelihood algorithm of JoinMap with population type CP; grouping at LOD>15; recombination fraction ≤0.4; ripple value = 1; jump in goodness-of-fit threshold (the normalized difference in goodness-of-fit chi-square before and after adding a locus) equal to 5 under a Kosambi mapping function. Marker ordering with JoinMap was carried out by simulated annealing, excluding markers that contributed to unstable marker orders in the first two ordering rounds to yield a higher likelihood support framework map. Additional segregating markers were then fitted to the linkage maps at lower stringency by the third and final round of JoinMap to provide map position for a larger number of segregating DArT markers.

### Comparative Analysis between the Linkage Map and the Assembled Genome Sequence

A genome-wide assessment of the consistency of the marker order estimated in the linkage map derived from this particular pedigree with the physical position of the markers in the currently assembled genome sequence was carried out by aligning the higher confidence framework linkage map to the 11 main scaffolds of the current assembly of the *E. grandis* genome sequence (version 1.0 available in Phytozome 6.0) produced for the one-generation selfed tree 'BRASUZ1' (Brazil Suzano S_1_). This alignment was also used to provide pseudochromosome-specific and genome-wide estimates of the correspondence between physical distance and recombination fraction in the *Eucalyptus* genome, as well as an estimate of the effective genome coverage provided by the framework map.

### Genomic Characterization of DArT Marker Probes

*E. coli* clones containing the 7,680 *Eucalyptus* DArT probes [25] were re-arrayed in twenty 384-deep-well-plates and submitted for bi-directional Sanger sequencing to the genomics facility of Purdue University (www.genomics.purdue.edu). Following quality trimming and clipping of vector regions and *Pst*I sites, sequences obtained were deposited in GenBank (accession numbers HR865291-HR872186). Redundancy of DArT probes at the sequence level was investigated using Geneious Pro 5.1.7 [49] using a minimum sequence overlap of 50 bp for a sequence to be assembled into a contig and an overlap identity of 98% (the “overlap identity” is the minimum percentage of bases that must be identical in the region of overlap in order for a sequence to be assembled). The numbers of unique and redundant DArT probes were then assessed by applying four different sets of sequence assembly parameters, from a most stringent assembly (A1) to the most liberal one (A4). These parameters were: (a) word length, i.e. the minimum number of consecutive bases that must match perfectly in order to find a match between two sequences; (b) maximum number or single base mismatches allowed per reads as a percentage of the size of the overlap between two reads; (c) maximum number of base ambiguities allowed in word matches; (d) maximum number of gaps that may be inserted into each read as a percentage of the size of the overlap between two reads; (e) maximum size of each gap that may be inserted into reads.

After preprocessing to remove contaminants, sequencing artifacts and low quality sequences, all DArT probes for which sequences were obtained were mapped to the assembled *Eucalyptus grandis* reference genome (version 1.0 available in Phytozome 6.0). Mapping was carried out using the BWA-SW (version 0.5.8) component from the Burrows-Wheeler Alignment tool [50] to produce a BAM [51] file. As the BWA tool can detect chimerical reads reporting two or more hits, parameters were set up such that non-optimal mapping was avoided. The threshold for a probe sequence hit to be retained was set to a fixed value (T = 70), corresponding to twice the median value of the numeric distribution obtained from the formula 5.5×log (L). This formula was applied to each one of the DArT probe sequences with length L, accounting for the fact that this formula is used by BWA as a coefficient for threshold adjustments. As a consequence, BWA did not search for suboptimal hits with a score lower than the alignment score minus T. The BWA options for the alignment score calculation including the score of a match (a), mismatch penalty (b), gap open penalty (q), and gap extension penalty (r) were left at their default settings (a = 1; b = 3; q = 5; r = 2). As an additional evaluation of the quality of the mapping procedure, sequence alignment information was extracted from the BAM file using an in-house Perl script designed to report all queried sequence hits and sub-optimal alignment scores. We considered up to two hits to be a “successful mapping” and used the sub-optimal scores for each one of the hits to classify the “mapping reliability” and “expected mapping error rate” of the procedure. Finally, to inspect the genomic features of the DArT markers relative to predicted gene models in version 1.0 of the *Eucalyptus grandis* genome, the 11 scaffolds were partitioned into 5 Mbp bins. A Spearman rank correlation between the number of DArT markers and number of gene models annotated in each bin was estimated for each pseudochromosome scaffold. Additionally the physical distance in base pairs from each sequenced DArT probe to the closest gene model was estimated to provide a genome-wide picture of the gene-space coverage of the *Eucalyptus* genome provided by the DArT array.

## Results

### DArT Marker Genotyping

The distribution of markers across the different levels of reproducibility was skewed towards the highest quality classes. For example, out of the 3,933 markers that had reproducibility ≥95%, 70% of them had reproducibility equal to or greater than 99%. For the 4,884 markers that passed the threshold quality score, 61% had Q ≥70, while out of the 5,415 markers with a call rate ≥75%, 36% had a call rate ≥90% (Figure S1). While reproducibility and Q score are measures that directly appraise the quality of the genotyping, the call rate essentially reflects the percent missing data tolerated. A relatively less stringent marker call rate of ≥75% was adopted to maximize the number of markers positioned on the linkage map, since such a threshold would still yield good quality marker data for ∼128 informative recombinant gametes that allow satisfactory marker linkage and ordering analyses during map construction. The 7,680 probe *Eucalyptus* microarray yielded a total of 3,191 markers that simultaneously passed all marker quality and call rate filtering parameters (Figure S1).

Out of 3,191 markers tested for Mendelian behavior only 215 did not fit either a 1∶1 or a 3∶1 ratio and were excluded from further analyses. Out of the 2,976 DArT markers that showed Mendelian behavior and were used in the linkage analysis, 1,777 segregated in a 1∶1 pseudo-testcross configuration and 1,199 in a 3∶1 fashion, i.e. were heterozygous loci segregating simultaneously from the two parents. Regarding the microsatellite dataset, 166 loci were fully informative with three or four alleles segregating in four distinct genotypic classes providing valuable anchor loci for the construction of an integrated linkage map. Forty-two microsatellites segregated from one of the parents only, 25 from *E. grandis* and 17 from *E. urophylla*, while 14 segregated in a 1∶2:1 F2 phase-unknown configuration.

### Linkage Mapping

**Figure 1:**
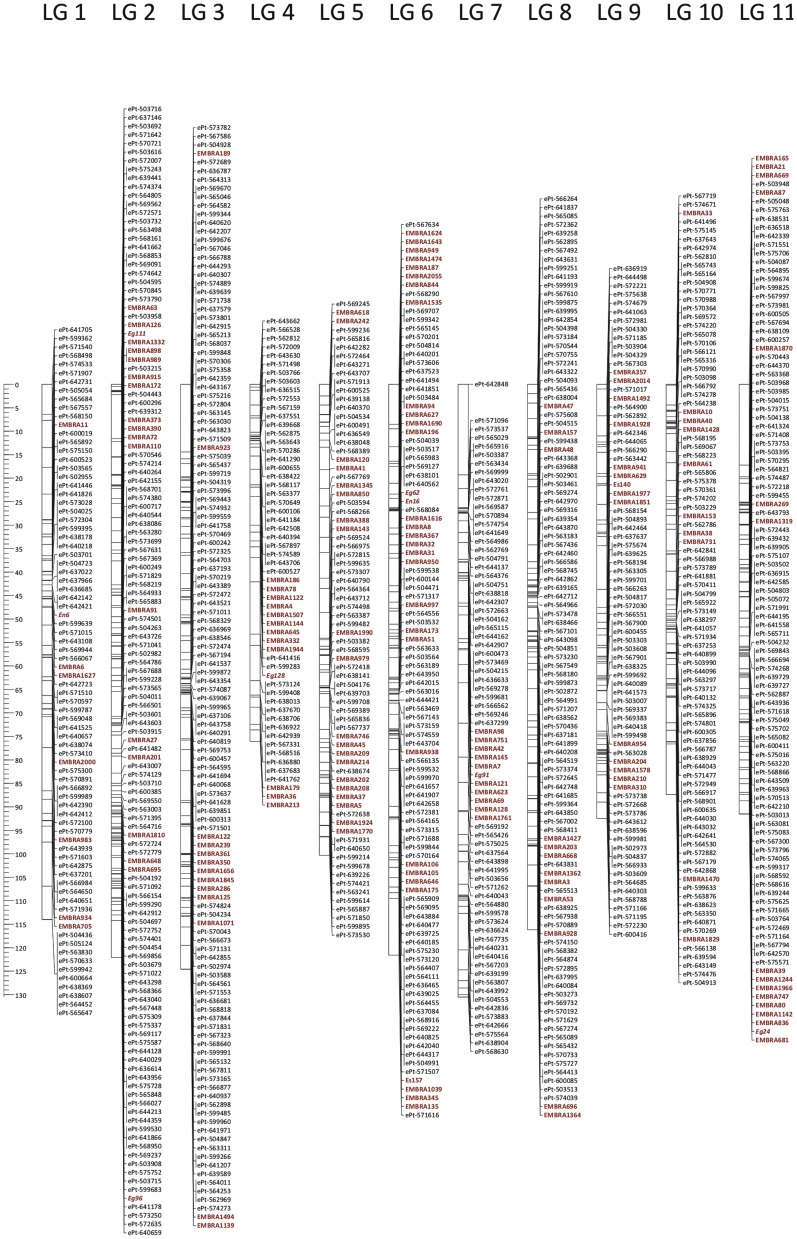
Framework DArT/microsatellite linkage map for *Eucalyptus*. The map includes 1,029 markers positioned with high confidence for locus order, involving 861 DArT (in black) and 168 microsatellites (in red) with a centiMorgan scale on the left.

A dataset with 3,198 markers (2,976 DArT and 222 microsatellites) was subjected to a mapping analysis. Grouping analysis at LOD>15.0 resulted in 2,980 markers grouped in 11 *bona fide* groups (numbered as established earlier [44]) and assessed by the presence of anchoring microsatellite markers. The linkage map built with higher likelihood support for marker order following the second round of JoinMap is presented as a “Framework map” (Figure 1). The linkage map obtained after the third round of ordering is hereafter called the “Full map” and is presented as a way to provide a preliminary position from which all the informative markers fed into the subsequent genomic characterization analysis (Table 1 and Figure S2). The Framework map, built following the second round of JoinMap, resulted in 1,029 markers positioned with higher confidence, 861 DArT and 168 microsatellites. When a more liberal marker ordering was allowed, a total of 2,484 markers were mapped (2,274 DArT markers and 210 microsatellites). The remaining 496 markers could not be mapped, even using a relaxed stringency, possibly as a result of redundancy of DArT markers at the sequence level (see below) or due to very close linkage so that not enough recombinants could be sampled to resolve relative ordering along the map.

**Table 1:** Mapping statistics of the DArT/microsatellite consensus maps of *Eucalyptus grandis x E. urophylla.*

Linkage Group/Pseudochromosome	1	2	3	4	5	6	7	8	9	10	11	Total	Mean	St.dev.
**Full map** a														
Total # markers	207	244	270	106	189	271	224	275	220	263	215	2,484	225.8	49.34
# DArT markers	191	219	256	93	166	231	210	262	204	246	196	2,274	206.7	47.68
# Microsatellites	16	25	14	13	23	40	14	13	16	17	19	210	19.1	7.98
Total size (cM)	167.8	129	102.4	86.6	130.7	116.5	117.3	118.6	118.3	117.4	99.5	1,303.9	118.5	20.8
Average inter-marker distance	0.8	0.5	0.4	0.8	0.7	0.4	0.5	0.4	0.5	0.4	0.5	0.5	–	–
**Framework Map** b														
Total # markers	80	131	128	57	74	104	75	107	78	92	103	1,029	93.5	23.4
# DArT markers	72	112	115	44	54	72	64	95	64	82	87	864	78.3	22.6
# Microsatellites	8	19	13	13	20	32	11	12	14	10	16	168	15.3	6.6
Total size in cM	114.0	122.1	124.6	76.1	100.3	121.6	130.5	116.2	92.5	87.4	91.5	1,176.8	107	18.1
Average inter-marker distance	1.4	0.9	1.0	1.3	1.4	1.2	1.7	1.1	1.2	1.0	0.9	1.1	-	0.3
**Framework to genome map** c														
Total # framework markers	62	98	102	52	68	88	68	94	66	82	89	869	79	16.5
Physical dist. covered (Mbp)	40.7	63.8	79.7	41.1	73.8	50.3	51.9	68.4	38.4	38.6	40.8	587.5	–	–
Ratio kbp/centiMorgan	357.3	522.1	639.2	539.9	736.1	413.7	548.4	588.9	415.1	441.4	445.4	–	513.4	112.7

a Full map: all markers mapped at relaxed support for order.

b Framework map: markers ordered with higher statistical support.

c Framework to genome map: framework markers were positioned onto the assembled *Eucalyptus grandis* genome sequence to provide a correspondence between physical distance and recombination fraction for each pseudochromosome and at the genome-wide level.

A much larger proportion of segregating microsatellites (80%) could be fitted in the Framework map than DArT markers (45%), most likely due to the higher information content of the fully segregating multiallelic microsatellites that provide higher power to categorize recombinant *versus* parental haplotypes and thus determine order. However, the final size of the Framework map was only 9.5% smaller than the Full map (1,176.7 versus 1,303.9 cM) (Table 1 and Figure S2). Marker orders of the Framework map and the Full map were generally consistent, although some inverted sets of markers were observed, mainly on linkage groups 1, 2, 7 and 9. Furthermore, although the expectation was that all framework markers would be contained in the Full map this was not always the case. The Framework map contained 99 markers that were excluded when a relaxed ordering threshold was allowed upon Full map construction. They were concentrated on linkage groups 2 (42 markers), 3 (26 markers), 1 (19 markers) and 11 (8 markers) (Figure S2).

The Full map of DArT markers contained on average 226 markers per linkage group positioned at a sub-centiMorgan average inter-marker distance of 0.5 while the Framework map had on average 93.5 markers per linkage group and yielded a 1.1 cM average inter-marker distance (Table 1). The distribution of map distances between consecutive markers in the Framework map was significantly different from the one in the Full map (p = 0.021 in a non-parametric Komolgorov-Smirnov test) (Figure S3). This result demonstrates that (i) a Framework map spreads out well-supported markers to provide robust locus ordering and (ii) reduces the proportion of short inter-marker distances (<1 cM) relative to a Full map (87% in the Full map and 65% in the Framework map).

A tally of the origin of the 861 DArT markers mapped on the Framework map showed that 197 (23%) markers segregated 1∶1 from *E. urophylla*, 298 (35%) from *E. grandis,* while 366 (42%) were heterozygous in both parents segregating 3∶1. Very similar proportions were observed when all 2,274 DArT markers were examined. These results suggest a higher sequence heterozygosity in the *E. grandis* parent than in the *E. urophylla* parent. Out of the 7,680 marker probes in the array, 2,274 (i.e. approximately 30%), were ultimately mapped in this single segregating family. However, if the 3,191 DArT markers that passed the genotyping quality filters for this experiment were considered, 71% of the markers could be mapped. Although the proportion of markers that can be mapped depends largely on the sequence heterozygosity of the parents and their genetic divergence, this result corroborates the outstanding performance of the DArT array for linkage mapping purposes in *Eucalyptus*.

### Recombination and Physical Distances in the *Eucalyptus* Genome

**Figure 2:**
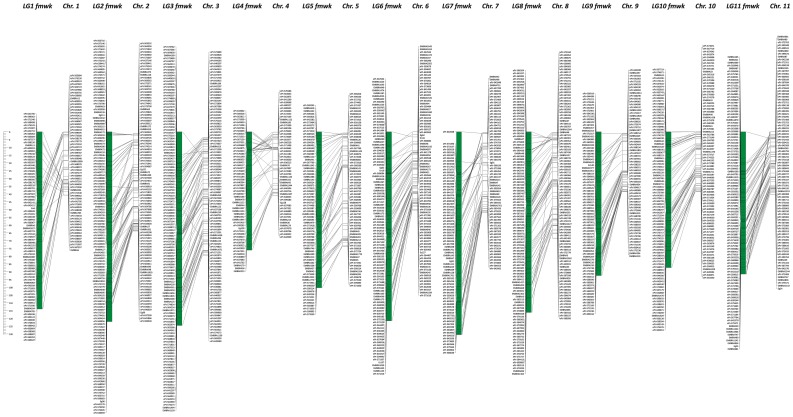
Alignment of the Framework map to the *Eucalyptus grandis* reference genome. Correspondence of the DArT and microsatellite marker positions on the Framework linkage map (green bars) with their location on the 11 *Eucalyptus grandis* pseudochromosome scaffolds (white bars). The scale on the left corresponds simultaneously to centiMorgan distances for the linkage map and to Mbp of sequence for the pseudochromosome scaffolds.

The alignment of the Framework linkage map to the *Eucalyptus* genome sequence indicates that the relative order of linkage mapped markers by and large agrees with their relative physical positions (Figure 2). Typically only a few sparse markers or small blocks of markers (e.g. LG1 and LG4) show a locally inconsistent order with the one estimated in the genome sequence. Upon further inspection of the segregation data of the few scattered markers showing discrepancy between their physical- and recombination-based positions, several of them were borderline in terms of marker quality and call rate parameters, which could possibly explain the observed inconsistencies. Out of the 1,029 framework-mapped markers, 869 could be positioned on the genome sequence while the remaining 160 either had no sequence available for mapping or mapped to the 4,941 smaller additional unanchored scaffolds of the current *Eucalyptus* genome assembly. The 869 linkage- and physically-mapped markers covered a total of 587.5 Mbp of sequence (Table 1) thereby providing 97% coverage of the 605.8 Mbp currently assembled in the 11 main scaffolds of the *Eucalyptus* genome. Pseudochromosome-specific estimates of the relationship between physical distance in kbp and recombination fraction in cM varied between 357.3 for pseudochromosome 1 and 736.1 for pseudochromosome 5, with a genome-wide average of 513.4 kbp/cM (Table 1). When the full linkage map was aligned to the genome sequence (data not shown), out of the 2,274 genetically mapped segregating DArT markers, 1,986 aligned to the 11 pseudochromosomes, while 45 markers mapped to 31 unanchored scaffolds and 243 DArT markers had no sequence available or did not map to the current assembly. Based on the linkage-mapped DArT markers, the 31 unanchored scaffolds, adding up 1.4 Mbp of sequence, could be assigned to the 11 main pseudochromosomes (Table S1).

**DArT probe sequence redundancy analysis.** Sequences were obtained for 6,918 of the 7,680 DArT probes (90%), with average size of 534 bp. Under the most stringent assembly parameters (see Material & Methods), out of the 6,918 sequences, 3,709 fell into multi-sequence clusters with two or more sequences per cluster. These were merged into 1,374 unique clusters of non-redundant sequences, while 3,209 sequences were unique, unmatched singletons. In total, the 6,918 probes for which sequences were obtained represented effectively 4,583 unique loci, i.e. a low bound estimate of the rate of redundancy of 33.75%. Under more liberal assembly parameters, the total number of unique loci was reduced to a total of 3,864, providing a high-end estimate of the rate of sequence redundancy at 44.14% (Table 2). If an equivalent rate of redundancy is assumed for the 762 DArT probes for which no sequences could be obtained, the 7,680 probes in the DArT array effectively sample between 4,289 and 5,087 unique loci in the *Eucalyptus* genome. All 6,918 sequences were submitted to GenBank and 6,896 were eventually accepted and deposited (22 were trimmed to sizes smaller than acceptable by the NCBI), receiving accession numbers HR865291-HR872186, with clone identifiers corresponding to the DArT marker naming convention used in this report. Searched against the complete NCBI EST database, 3,703 (53.6%) returned with positive BLASTn hits (Table S2).

### DArT Marker Probe Alignment to the *Eucalyptus* Genome

Out of the 6,896 DArT probes for which quality sequences were obtained, 6,631 (96%) could be successfully aligned to the assembly of the *Eucalyptus grandis* genome sequence (version 1.0 in Phytozome 6.0) while 265 DArT probes could not be mapped using high stringency parameters. Of the mapped probes, 6,390 of them aligned to the 11 main pseudochromosomes and 241 to the additional 4,941 small unanchored scaffolds. When these mapping results were used to assess the quality of the sequence alignment parameters adopted (see Material and Methods) a mapping error rate of 0.002 was estimated by observing 12 unsuccessfully mapped sequences in 6,631, i.e. a reliability ≥99.8%. Interestingly, this number matches precisely the average scoring reproducibility estimated by DArTsoft following the standard marker selection thresholds used. When the threshold for a probe sequence hit to be retained was set down to the BWA default level (T = 37), 166 of the 265 unmapped probes could be additionally aligned to the 11 main pseudochromosomes and 91 of these 166 probes were also linkage-mapped onto the Full map. Additionally, out of the 89 probes that remained physically unmapped to the *E. grandis* genome assembly, 36 were successfully linkage-mapped as well.

A further examination of the alignment of the 6,631 DArT probes to the *Eucalyptus* genome assembly, including all 4,952 scaffolds, was carried out. A total of 4,189 probes were confidently aligned to a single and unique position in the genome as their mapping produced a single hit with no subalignment score. For 2,252 of the 2,442 remaining probes, a second subalignment was retained which overlapped the same locus as the one of the first best alignment. Therefore in total 6,441 DArT probes out of the 6,631 evaluated (97.1%) were considered to be aligned to a single locus in the genome. For the 190 probes in which a second hit was reported by BWA, we carried out an analysis using chimeric tools detection provided by the CD-HIT [52] and EULER DNA [53] fragment assembly softwares with default parameters, resulting in no detectable chimeric reads. For 135 probes, the retained subalignment was located in a different position on the same scaffold, and in 95 of these cases the distance between alignments was smaller than 1 kb, suggesting a contiguous tandem duplication. For 40 DArT probes the distances between the first and second hit alignments were larger than 1 kb, with 13 of them larger than 10 kb. Finally, only 55 probes were aligned to positions on different pseudochromosomes. In 37 of these cases both loci were found in the 11 main pseudochromosomes and in 18 cases one of the loci was found in an unanchored scaffold. The frequency of multilocus DArT probes in different chromosomes observed in *Eucalyptus* (55 in 6,631, i.e. 0.83%) is consistent with the 1.4% frequency observed in a linkage mapping study in barley [54].

### DArT Marker Coverage of the *Eucalyptus* Gene-space

**Figure 3:**
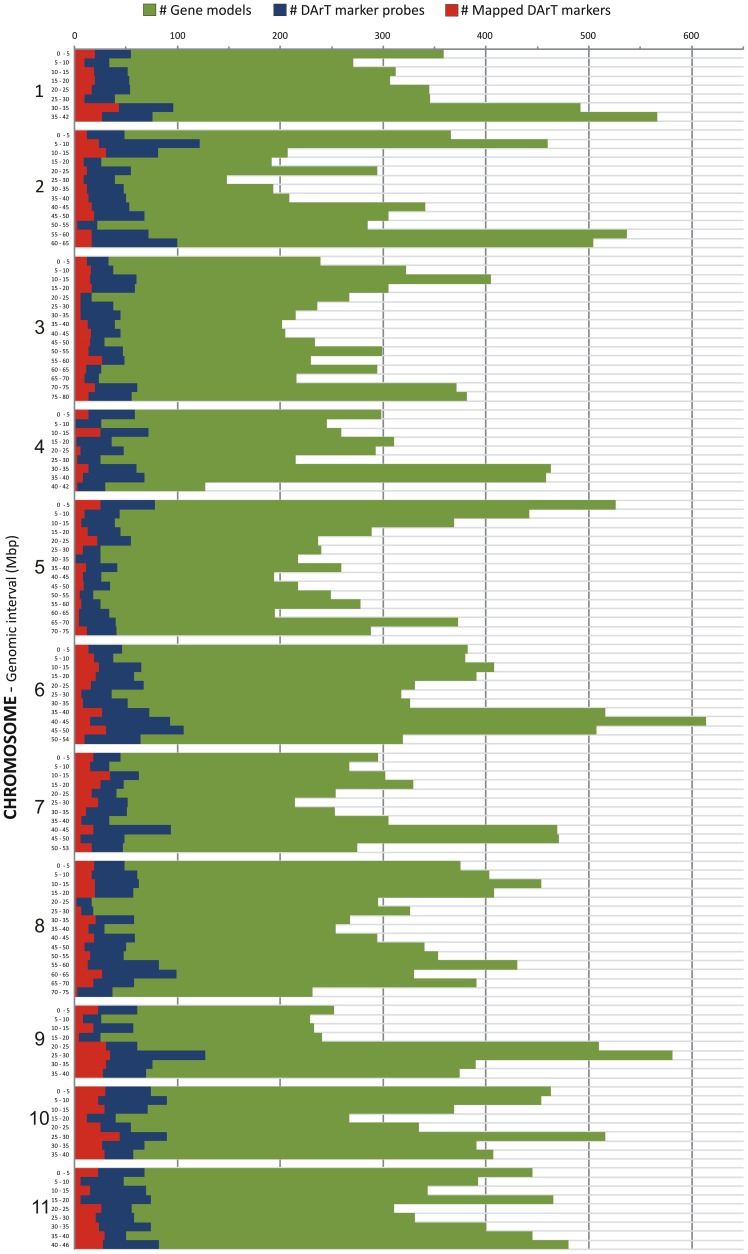
Genome-wide correspondence of DArT markers and predicted gene models in the *Eucalyptus grandis* genome. The 11 pseudochromosomes of the *Eucalyptus grandis* genome (Version 1.0 in Phytozome 6.0), were partitioned into 122 bins of 5 Mbp. For each bin the numbers of DArT marker probe positions (blue bars), the number of genetically mapped DArT markers (red bars) and the number of predicted gene models (green bars) were plotted.

**Figure 4:**
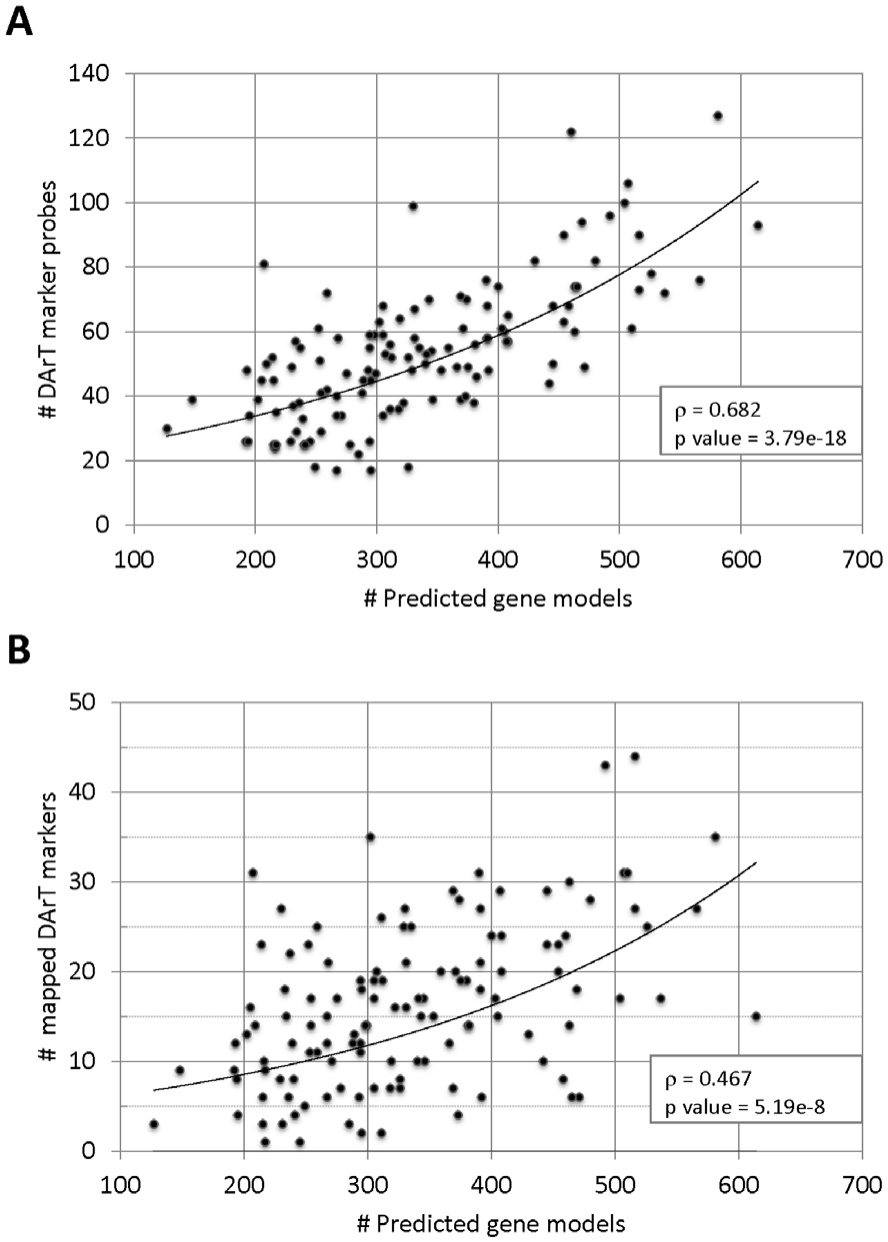
Correlations between DArT markers probes, mapped DArT markers and gene models. Spearman Rank correlations were estimated between: (A) the number of DArT marker probes and the number of gene models; and (B) the number of mapped DArT markers and the number of gene models, for every 5 Mbp genome bin.

**Figure 5:**
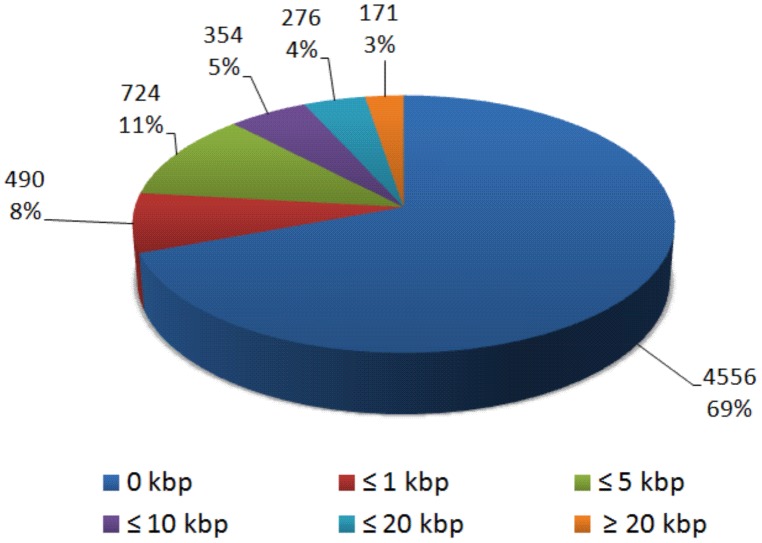
Distribution of the physical distance between DArT markers and gene models in the *Eucalyptus* genome. Distribution of the proportions of the 6,571 DArT marker probe positions according to distance classes in kbp from the closest predicted gene model in the *Eucalyptus grandis* genome (version 1.0).

To characterize the *Eucalyptus* gene-space covered by the DArT array, we considered only the probes that were aligned to the 11 annotated pseudochromosomes. These totaled 6,390 probes which aligned to a total of 6,571 positions, given that 181 probes also aligned to a second position according to the BWA threshold adopted. The distribution of both the 6,571 DArT probes positions and the 1,986 genetically mapped DArT markers, were plotted together with the distribution of the 41,204 predicted gene models in the *Eucalyptus* genome (version 1.0) partitioned into 122 bins of 5 Mbp each, which on average correspond to ∼10 cM map distance bins, assuming a ∼1200 cM total recombination distance (Figure 3). The histogram indicates that the DArT microarray provides a homogeneous genome-wide coverage of markers and suggests a monotonic relationship between the number of gene models and the number of DArT markers. In fact a relatively strong and highly significant Spearman rank correlation was found between the number of predicted gene models and the total number of DArT markers found in a genome bin (ρ = 0.682; p = 3.79e-18), and likewise with the number of mapped DArT markers (ρ = 0.467; p = 5.19e-8) (Figure 4). These results show that the DArT array tends to provide segregating markers in essentially all 5 Mbp genomic bins with the number of DArT markers scaling with the number of genes in the bin. On average each bin contains 16±9.0 genetically mapped markers, 53±21.6 DArT marker probes and 334±100.9 predicted gene models. Only four bins had fewer than 20 DArT marker probes mapping to them and only 11 out of the 122 bins had fewer than 5 genetically mapped markers. In addition, almost 70% of the DArT marker sequences were mapped at zero bp from the closest predicted gene model and less than 10% were located further than 10 kbp from predicted gene models (Figure 5).

## Discussion

This study provides unprecedented data regarding the detailed genomic attributes of DArT markers in a plant genome. Following the development of a high performance *Eucalyptus* DArT array [25] we have now examined the genomic properties of the DArT marker probes that populate this array by sequencing them, constructing a high density linkage map and carrying out physical comparisons to the recently released *Eucalyptus grandis* BRASUZ1 annotated genome sequence. We have shown that DArT marker probes preferentially target the gene space and display a uniform distribution across the genome, providing excellent coverage for genome-wide applications in breeding and diversity studies. Such ubiquitous DArT marker properties had not been described previously in spite of several DArT marker-based applied studies published to date for a large number of plant species.

### DArT Marker Genotyping Efficiency for Genetic Analysis in *Eucalyptus*

A set of relatively strict filtering parameters was applied to the signal intensities obtained from the 7,680 probes in the *Eucalyptus* microarray. A total of 3,191 markers (41.5%) passed all marker quality and call rate thresholds (Figure S1), a proportion consistent with the original estimates reported during array validation [25] and recent mapping studies in similar interspecific pedigrees [29], [30]. Besides marker quality filtering, a strict screening for adherence to Mendelian expectations was applied. Segregation distortion has been reported in some previous *Eucalyptus* mapping studies although, most of the time, at a rate no different from the one expected by chance alone [34], [44]. This became a topic of interest as a way to assess heterospecific interactions in the F1 hybrid affecting introgression rates between distant species [55]. In this particular pedigree, however, just as in the first linkage map study in *Eucalyptus* [34], no segregation distortion would be expected in principle, since marker segregation was observed from each pure species parent and not in the gametes derived from a F1 hybrid where distortion could be expected. Accordingly, only 215 markers (6.7%) were excluded due to departures from expected segregation ratios, a proportion close to the 5% expected by chance alone. Besides sampling error, these distorted markers could include cases of duplicated loci such as the 55 DArT probes shown to confidently align to positions on different pseudochromosomes, and the 13 probes aligning at distances greater than 10 kbp, which in both cases would give rise to mixed hybridization signals and distorted segregation ratios. The 97% genome-wide physical coverage provided by the linkage map built in this study indicates that keeping distorted markers in the linkage analysis would not improve coverage but, rather, could complicate marker ordering if distortions derived from excessive missing data or mistyping were included [56].

Out of the 2,976 DArT markers included in the linkage analysis 2,274 were eventually mapped and ordered in the full consensus map and 1,029 in the framework version (Table 1). The proportion of DArT markers mapped is well within the predicted number of useful markers for mapping, between 1,818 and 2,553, as originally reported when the *Eucalyptus* DArT array was developed [25]. This number is also comparable to the 2,229 DArT markers mapped in the consensus map of two related backcross families of *E. grandis x E. urophylla* [30] and to the 1,845 EST-based Single Feature Polymorphism markers map reported earlier for the same pedigree used in this study [42]. The proportion of informative markers in such interspecific mapping pedigrees has been considerably higher than the number observed in intraspecific pedigrees. Hudson et al. [29] could only map 1,060 DArT markers in an outcrossed F2 family derived from two inter-provenance F1 individuals and only 569 in an inter-provenance cross of *E. globulus.* Genetic divergence between species and corresponding levels of differential sequence heterozygosity at the DArT loci determine in large part the proportion of informative markers ultimately captured. The DArT genotyping platform has provided an order of magnitude larger number of markers for mapping in *Eucalyptus* than previous technologies such as RAPD, AFLP and microsatellites [32]. The undomesticated nature of *Eucalyptus* resulted in a larger number of markers mapped than most DArT-based linkage maps built with extensively optimized DArT arrays such as those for wheat, oats [23], sorghum [57], and barley [54].

Recently, between 3,100 and 3,500 high quality polymorphic DArT markers were scored in breeding populations composed of several hundred individuals of *E. grandis* and *E. urophylla* in the context of Genomic Selection experiments [28]. As expected, when compared to an average of 2,200 to 2,300 markers captured and mapped in biparental pedigrees, the DArT array provides between 40 and 50% more markers at the population level. If similar proportions are kept, one can anticipate that in *E. globulus* breeding populations, the DArT array will provide between 750 and 1,500 informative markers depending on the general variability of the population and provenance composition.

### Probe Redundancy is a useful Property of the DArT Array

Reported estimates of DArT marker redundancy obtained by comparing the segregation pattern in mapping population or estimating Hamming distances between markers have varied from 38% in barley [54], to 43% in *Arabidopsis* [58]. After sequencing all DArT probes on the array, a redundancy of between 33.75 and 44.14% was estimated (Table 2). This is consistent with the 46% redundancy rate reported for several thousand sequenced DArT probes from an oat genotyping array [23]. Under the same genome complexity reduction protocol, redundancy will vary with the particular genome structure of the target species, the diversity of samples used to build genomic representations and, largely, with the probe screening criteria and the final number of selected probes. As more probes are surveyed, a higher redundancy will result. Redundancy from sequencing only a few hundred DArT probes, previously selected for polymorphism, was estimated at 15% in an apple array, although a potential redundancy of 50% was acknowledged had all clones on the array been sequenced [24]. Considering that a fairly uniform physical and mapping distribution of the DArT probes was achieved across the *Eucalyptus* genome (Figures 2 and 3), a certain level of probe redundancy in the DArT array is actually a desirable feature. *Eucalyptus* DArT probes vary in size (534±215 bp) and, although sharing portions of DNA sequence, will have variable abilities to detect sequence polymorphism across individuals and populations, thereby providing improved power and flexibility for genome-wide genotyping.

**Table 2:** Results of redundancy analysis of the 6,918 DArT marker probe sequences under four different sets of assembly parameters from the most stringent (A1) to the most relaxed (A4) (see Material and Methods for details).

Parameter	A1	A2	A3	A4
Word length	18	14	12	10
Index Word length	13	12	11	10
Mismatches	10%	15%	20%	20%
Ambiguities per read	4	4	16	16
Maximum % gaps per read	10%	15%	20%	20%
Gap size	1bp	2bp	5bp	5bp
**Results of redundancy analysis**				
# Unmatched singleton^a^	3,209	2,607	2,381	2,276
# Redundant sequences^b^	3,709	4,311	4,537	4,642
# Unique non-redundantsequences^c^	1,374	1,537	1,587	1,588
Total selected sequences^d^	4,583	4,144	3,968	3,864
Estimated rate of sequenceredundancy	33.75%	40.0%	42.64%	44.14%

a Unique sequences not matching any of the other reads.

b Sequences that fall into multi-sequence clusters with more than two sequences per cluster.

c Unique sequences drawn from the redundant clusters.

d Sum of unmatched singleton and non-redundant sequences.

An interesting aspect of the DArT probe sequence redundancy emerged when comparing the alignment of DArT probes to the reference genome. Between 3,864 and 4,583 unique sequences were observed for the DArT probes (Table 2), an estimate consistent with the 4,189 DArT probes confidently aligned to a unique position in the genome. Nevertheless the BWA alignment to the genome assembly revealed that 2,252 additional probes mapped to exclusive positions so that, in total, 6,441 loci in the genome were sampled by the DArT array. This is the first time such an analysis has been carried out for a species for which a DArT array and a reference genome are available. It shows that redundancy estimates based on a simple assembly of DArT probe sequences tend to be conservative.

### Framework Linkage Mapping Allows Reliable Estimates of the kbp/cM Ratio in the *Eucalyptus* Genome

Two versions of a linkage map were built in this study, each one with specific objectives. A Framework map was built as the "hypothesis" that best explained the segregation data observed, to provide more accurate information regarding marker order [59], [60] and to be used for the estimation of the relationship between physical distance and recombination fraction (Figure 1). On the other hand, the Full map, that included all segregating DArT markers, was built to provide a preliminary position for all possible DArT markers and thus allow a more extensive assessment of the genome coverage and distribution of DArT markers relative to genes. Additionally, by including the largest number of markers (even if at a relaxed order), this map offered a better probability of assigning unanchored scaffolds to the assembled pseudochromosomes of the current *Eucalyptus* reference genome sequence. Marker order of the Framework map and the Full map were generally comparable and total map sizes were also close (Figure S2 and Table 1). However, inverted sets of markers were observed between these map versions as well as markers that dropped out when going from one map to the other and *vice versa*. These results substantiate the well-known fact that the resolution of marker order is not a trivial issue and more so when a large number of markers are mapped with a limited progeny size [61]. This observation also supports the fact that similar apparent inversions and non-colinearities reported in previous comparative linkage mapping studies across sexually compatible *Eucalyptus* species [29] are, by and large, ordering inconsistencies due to various sources of experimental error [56] and rarely should be taken as evidence of any relevant biological genomic occurrence unless independent validation data is available. With the availability of a reference genome for *Eucalyptus*, coupled to high throughput sequencing technologies and powerful assembly procedures, such validations might now become possible.

A simple visual inspection of the aligned Full and Framework maps (Figure S2) and the significant difference found between the distributions of map distances between consecutive markers in the two map versions (p = 0.021) (Figure S3), support the conclusion that framework map building largely removes highly clustered sets of DArT markers, leaving a sparser map with essentially equivalent genome coverage and improved statistical support for relative ordering. Moreover, when the linkage map of microsatellites and DArTs was compared with a microsatellite-only map, the total recombination distance did not change (data not shown). This additional result suggests that, while the microsatellites do provide adequate genome coverage, the DArT markers effectively cover the genome in previously unsampled genomic regions, thereby providing the necessary marker density for high-resolution mapping and genome-wide studies. The Framework map should therefore be taken as the most reliable map when it comes to comparative analysis with the reference genome or map-based efforts (such as looking for the co-localization of genes with potentially large effect QTLs).

The alignment and physical mapping of 869 framework mapped DArT and microsatellite markers to the 11 main scaffolds of the *Eucalyptus* genome sequence allowed an estimation of the relationship between physical distance and recombination fraction in each pseudochromosome and for the whole genome. This estimate varied considerably (357 to 736 kbp/cM) with a genome-wide average of 513 kbp/cM (Table 1). Interestingly, this estimate is not far from the coarse estimates of 395–559 kbp/cM reported early on, based on the first available linkage maps [34] and the first estimates of *Eucalyptus* genome size [62]. This time, however, by using the assembled genome sequence to which framework markers were mapped physically, an improved estimate was possible. Kullan et al. [30] using a different approach, based exclusively on a selected set of 153 pairs of flanking markers mapped at approximately 1 cM distance, estimated 633 kbp/cM. Besides the potential bias introduced by specifically selecting pairs of markers and, as a consequence, precluding the intrinsic variation in recombination *versus* physical distance along the genome, that estimate was based on markers ordered at a relaxed likelihood. We therefore consider the estimates presented in this work, both at the pseudochromosome level and whole-genome average to be better approximations for *Eucalyptus* (Table 1), although we acknowledge that recombination rates are expected to vary by orders of magnitude across a genome [63].

### Linkage to Physical Mapping Suggests a Pan-genomic Feature of the DArT Array and Completeness of the *Eucalyptus* Genome Assembly

The overall consistency between the order of framework mapped DArT markers and their physical order in the genome sequence substantiate the quality of scaffold assembly in the current *Eucalyptus* genome sequence (Figure 2). While the linkage map reported by Kullan et al. [30] was used effectively to assist scaffold ordering during genome assembly (J. Schmutz pers. comm.) the linkage map presented herein was not, and thus constitutes an independent validation of the current *Eucalyptus* genome. Furthermore, from the completeness standpoint, only 45 markers out of 2,274 linkage mapped ones could not be aligned to the 11 main scaffolds. Conversely, only 89 probes remained physically unmapped to the genome sequence. Although these unmapped markers could imply missing sections in the genome assembly, they could also correspond to sequences that do not exist in the *E. grandis* genome, recalling that the 7,680 probes on the array were developed from 18 genomic representations involving 64 different *Eucalyptus* species with a broad phylogenetic diversity. A scrutiny of the original source of these 89 unmapped DArT probes revealed that 65 of them came from genomic representation libraries built with DNA from species other than *E. grandis*, while 24 came from *E. grandis* [25]. Nevertheless we observed significantly more non-*E. grandis* DArT probes not mapping to the genome than what would be expected due to chance alone (Pearson Chi-square 5.03; p value = 0.0249). This result, together with the excellent performance demonstrated for diverse phylogenetic investigations in the genus [22], suggests a distinctive pan-genomic attribute of this *Eucalyptus* DArT array. While most probes correspond to core genomic features common to all individuals and *Eucalyptus* species, a few probes may be derived from the “dispensable genome” composed of partially shared and/or non-shared DNA sequence elements among species [64], [65].

Completeness of the current assembly was also supported by the observation that, out of 85.4 Mbp of unanchored sequence in 4,941 small scaffolds, only 1.4 Mbp across 31 scaffolds was captured by 45 mapped markers and all, but a couple, were located in intermediate positions along the linkage groups and not at the extremes (Table S1). Were the genome assembly incomplete, one would expect to capture a considerably larger proportion of unanchored scaffolds and sequence. In fact, during the poplar genome assembly, Drost et al. [66], using a medium-density 608 marker map, were able to anchor 116 sequence scaffolds to unique genetic positions in linkage groups, thereby adding to the genome some 35.7 Mbp of sequence out of the 75 Mbp still unanchored at the time. These results, together with the fact that 86% of the 4,941 unanchored *Eucalyptus* genome scaffolds are less than 20 kbp in length, strongly suggest that the vast majority of unanchored scaffolds correspond to fragments of alternative haplotypes of already assembled pseudochromosomes, possibly derived from regions of high heterozygosity in the *Eucalyptus* genome and not to missing portions of the genome.

### The *Eucalyptus* DArT Array Provides Uniform Genome-wide Coverage While Preferentially Targeting Gene-rich Regions

Results from BLAST hits and genome-wide analysis of the *Eucalyptus* DArT probe sequences (Table S2 and Figure 3) corroborated previous studies in other plant species reporting that *PstI*-based DArT markers are predominantly located in low-copy, gene-rich regions of the genome [23], [54], [67]. However, the opportunity to map the DArT probes to a fully annotated reference genome beyond a simple BLAST analysis against ESTs, revealed a highly significant relationship between the numbers of DArT markers and predicted gene models (Figure 4) with a small proportion of DArT probes located more than 10 kbp from the closest gene (Figure 5). This result is significant as it might help explain the unprecedented level of resolution that the DArT array has provided for population genetic and breeding studies across the full range of *Eucalyptus* species [22], [28]. Based on the genomic characterization of the DArT probe sequences reported in this study, phylogenies or population genetic surveys based on DArT markers can now be further explored according to the gene proximity or gene content of particular markers sets. Alternatively, DArT markers from specific genes or selectively neutral regions can be selected *a priori* for targeted phylogenetic reconstructions. Moreover, the combination between genome-wide coverage and predominant association to the gene-space could also account for the good performance of the DArT array in providing markers for accurate genome-wide predictive models in recent Genomic Selection (GS) studies [28]. It might now be possible to correlate the genomic attributes of the DArT markers to their specific contributions to the predictive ability of GS models or to the resolution of specific phylogenies and hence look for specific markers or genomic segments of particular interest in subsequent studies.

### Conclusion

The results of this work, following the recently published DArT-based genetic studies in *Eucalyptus* [22], [28], [29], [30], further highlight the value of this genotyping platform for genetics, breeding and evolutionary genome-wide surveys in species of this genus. Given the commonality of the methods used in developing DArT arrays, the genomic properties of the markers described in this study are likely ubiquitous to most if not all angiosperm plant genomes. The DArT technology has now evolved by taking advantage of high-throughput short read sequencing [68]. By combining its long time established genome complexity reduction method, also adopted by recently described genotyping-by-sequencing (GbS) protocols [69], [70], a considerable leap in genome-wide polymorphism detection has taken place. Nevertheless, the general genomic attributes of the GbS-derived markers as far as genome coverage and preferential targeting of gene-rich regions should remain essentially the same as those described in this study, although a much larger number of markers based on digital sequence counts rather than analog microarray signal are obtained, in addition to the scoring of co-dominant SNP markers. This advance might push down current costs of large scale high-throughput plant genotyping even further than the DArT and SNP platforms did in the last few years. However, the necessary informatics infra-structure required to handle, store and analyze the huge sequence files generated by GbS for several thousand samples will not be immediately available in the realm of most plant genetic resources and breeding operations. Microarray-based DArT genotyping with its standardized processing and analysis protocols shall therefore continue to be a useful tool for a number of applications in plant genetic analysis, particularly those that not necessarily require very high density genome-wide genotyping.

## Supporting Information

Figure S1 **Distributions of the number and percentages of DArT markers that passed the filtering thresholds adopted for reproducibility (≥95%), quality score (Q ≥65) and call rate (≥75%).** A Venn diagram consolidates the information showing all possible classifications of the DArT markers according to the three filtering criteria adopted. Only markers that satisfied simultaneously all three criteria were used for linkage mapping.
(PDF)

Figure S2 **Alignment of the Full map (yellow bars) to the Framework (Fmwk) map (green bars) for the eleven** ***Eucalyptus*** **pseudochromosomes built using JoinMap 3.0, showing the connections between the same loci on both maps.** The Full map includes a total of 2,484 markers, 2,274 DArT and 210 microsatellites while the Framework map has 1,029 markers positioned with higher confidence for locus order, 861 DArT and 168 microsatellites. DArT markers in black and microsatellites in red; centiMorgan scale on the left.
(PDF)

Figure S3 **Frequency distributions of Kosambi recombination distances between consecutive markers across the two linkage map versions.** The distribution of map distances in the Framework map was significantly different from the one in the Full map (p = 0.021 of a non-parametric Komolgorov-Smirnov test), confirming the fact that a Framework map spreads out the retained markers with high support for ordering and reduces the proportion of inter-marker distances smaller than one centiMorgan from a total of 87% in the Full map to 65% in the Framework map.
(PDF)

Table S1 List of the 45 DArT markers linkage mapped to the eleven groups but aligning to small unanchored scaffolds of the current *Eucalyptus grandis* genome assembly (version 1.0 into Phytozome 6.0). These linkage mapped DArT markers allowed the assignment of 31 small scaffolds (1.4 Mbp of total sequence) to the 11 main pseudochromosomes.
(PDF)

Table S2 BLASTn hits of DArT marker probes (Genbank accession numbers HR865291-HR872186) searched against the complete NCBI EST database (August 12 2010 build); 3,703 (53.6%) returned with positive BLASTn hits (e value <1e–5).
(XLSX)
