# Supplementary material for: Genomic Characterization of DArT Markers Based on High-Density Linkage Analysis and Physical Mapping to the Eucalyptus Genome
Source: PLoS One. 2012 Sep 11;7(9):e44684. doi: 10.1371/journal.pone.0044684 (PMC3439404; doi:10.1371/journal.pone.0044684)
Supplement: Figure S1 — Distributions of the number and percentages of DArT markers that passed the filtering thresholds adopted for reproducibility (≥95%), quality score (Q ≥65) and call rate (≥75%). A Venn diagram consolidates the information showing all possible classifications of the DArT markers according to the three filtering criteria adopted. Only markers that satisfied simultaneously all three criteria were used for linkage mapping. (PDF) [file pone.0044684.s001.pdf]

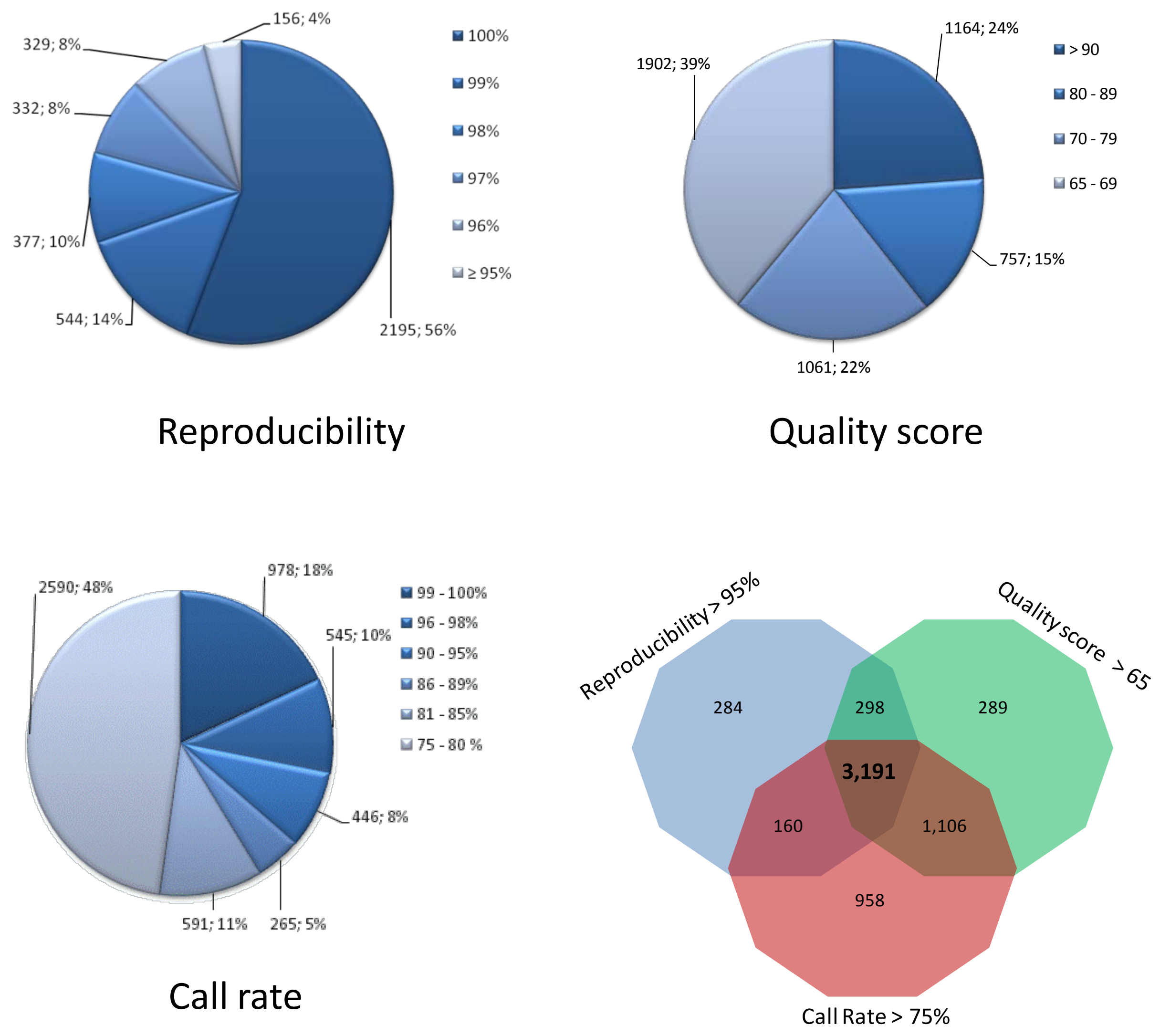

Figure S1. Distributions of the number and percentages of DArT markers that passed the filtering thresholds adopted for reproducibility (≥95%) quality score (Q ≥65) and call rate (≥75%). A Venn diagram consolidates the information showing all possible classifications of the DArT markers according to the three filtering criteria adopted. Only markers that satisfied simultaneously all three criteria were used for linkage mapping.
