# Supplementary material for: Genomic Characterization of DArT Markers Based on High-Density Linkage Analysis and Physical Mapping to the Eucalyptus Genome
Source: PLoS One. 2012 Sep 11;7(9):e44684. doi: 10.1371/journal.pone.0044684 (PMC3439404; doi:10.1371/journal.pone.0044684)
Supplement: Figure S2 — Alignment of the Full map (yellow bars) to the Framework (Fmwk) map (green bars) for the eleven Eucalyptus pseudochromosomes built using JoinMap 3.0, showing the connections between the same loci on both maps. The Full map includes a total of 2,484 markers, 2,274 DArT and 210 microsatellites while the Framework map has 1,029 markers positioned with higher confidence for locus order, 861 DArT and 168 microsatellites. DArT markers in black and microsatellites in red; centiMorgan scale on the left. (PDF) [file pone.0044684.s002.pdf]

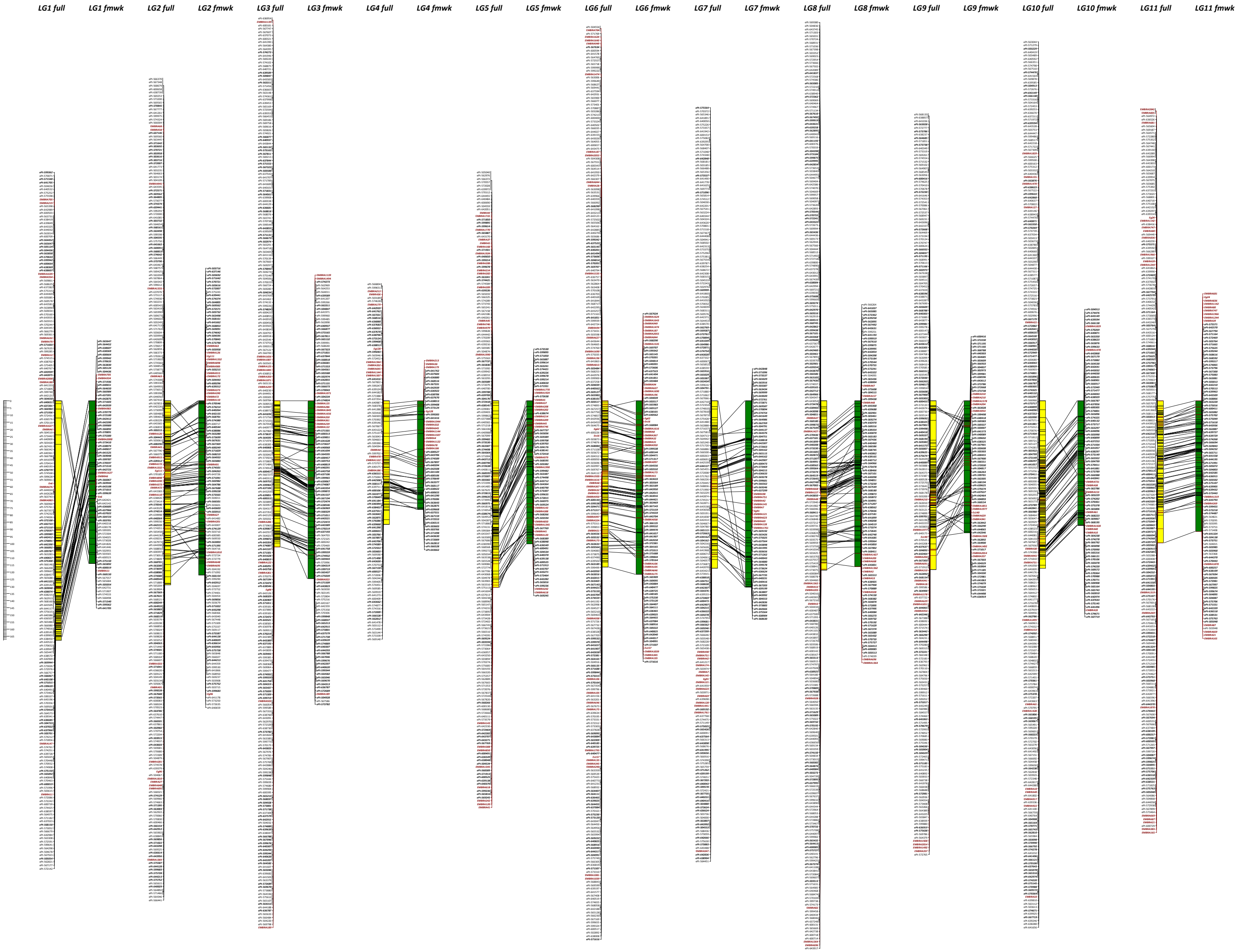

Additional File 2. Alignment of the Full map to the Framework (Fmwk) map for the eleven *Eucalyptus* pseudochromosomes built using JoinMap 3.0, showing the connections between the same loci on both maps. The Full map includes a total of 2,484 markers, 2,274 DArT and 210 microsatellites while the Framework map has 1,029 markers positioned with higher confidence for locus order, 861 DArT and 168 microsatellites. DArT markers in black and microsatellites in red; centiMorgan scale on the left.
