# Supplementary material for: Genomic Characterization of DArT Markers Based on High-Density Linkage Analysis and Physical Mapping to the Eucalyptus Genome
Source: PLoS One. 2012 Sep 11;7(9):e44684. doi: 10.1371/journal.pone.0044684 (PMC3439404; doi:10.1371/journal.pone.0044684)
Supplement: Figure S3 — Frequency distributions of Kosambi recombination distances between consecutive markers across the two linkage map versions. The distribution of map distances in the Framework map was significantly different from the one in the Full map (p = 0.021 of a non-parametric Komolgorov-Smirnov test), confirming the fact that a Framework map spreads out the retained markers with high support for ordering and reduces the proportion of inter-marker distances smaller than one centiMorgan from a total of 87% in the Full map to 65% in the Framework map. (PDF) [file pone.0044684.s003.pdf]

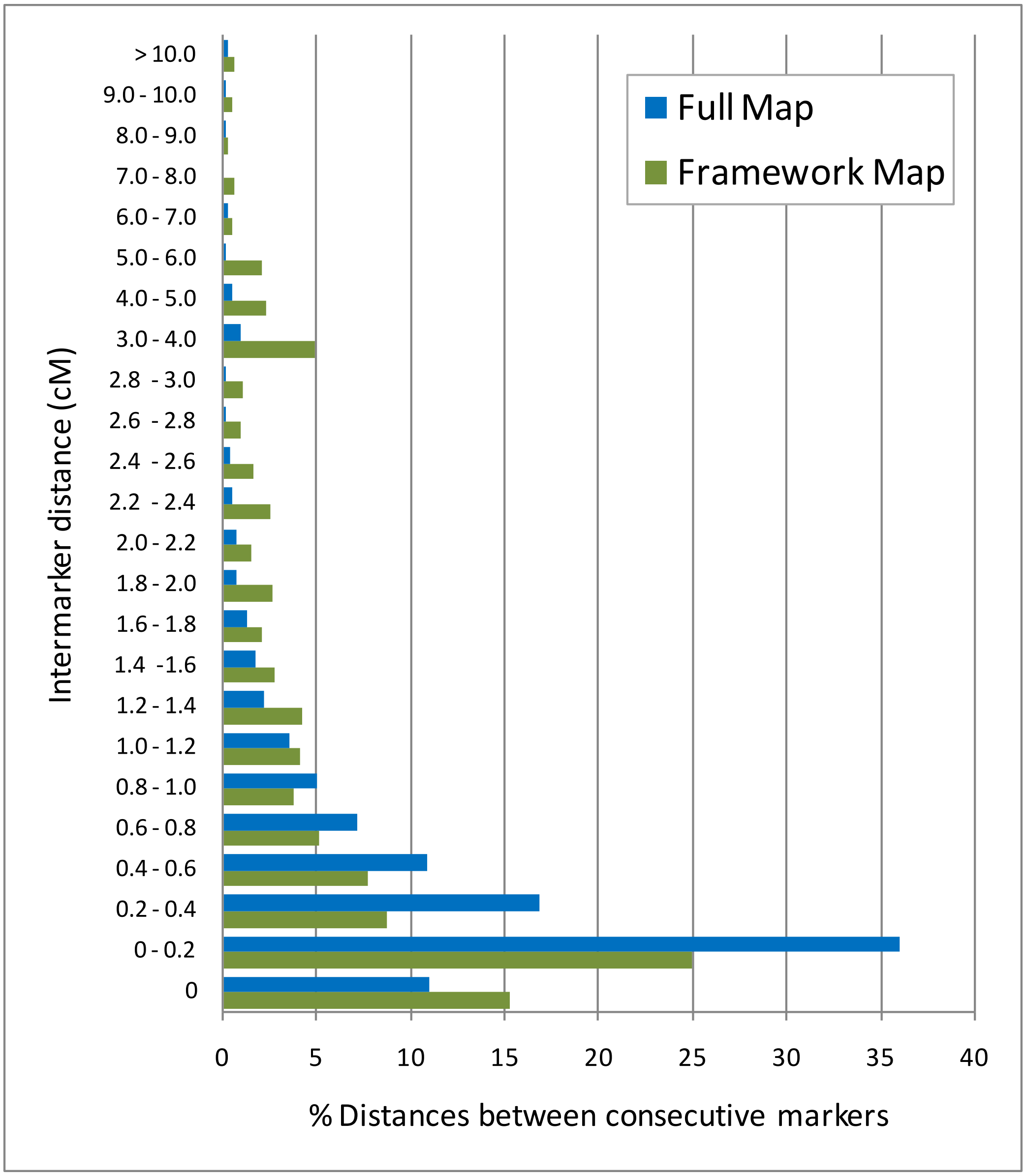

Figure S3. Frequency distributions of Kosambi recombination distances between consecutive markers across the two linkage map versions. The distribution of map distances in the Framework map was significantly different to the one in the full map (p = 0.021 of a non-parametric Komolgorov-Smirnov test), confirming the fact that a framework map spreads out the retained markers with higher support for ordering and reduces the proportion of inter-marker distances smaller than one centiMorgan from a total of 87% in the Full map to 65% in the Framework map.
