# Supplementary material for: Genomic Characterization of DArT Markers Based on High-Density Linkage Analysis and Physical Mapping to the Eucalyptus Genome
Source: PLoS One. 2012 Sep 11;7(9):e44684. doi: 10.1371/journal.pone.0044684 (PMC3439404; doi:10.1371/journal.pone.0044684)
Supplement: Table S1 — List of the 45 DArT markers linkage mapped to the eleven groups but aligning to small unanchored scaffolds of the current Eucalyptus grandis genome assembly (version 1.0 into Phytozome 6.0). These linkage mapped DArT markers allowed the assignment of 31 small scaffolds (1.4 Mbp of total sequence) to the 11 main pseudochromosomes. (PDF) [file pone.0044684.s004.pdf]

Additional File 4. List of the 45 DArT markers mapped to the eleven linkage groups and positioned onto small unanchored scaffolds of the current *Eucalyptus grandis* genome assembly available into Phytozome 6.0. These linkage mapped DArT markers allowed assigning 31 small scaffolds (1.4 Mbp of sequence) to the 11 main pseudochromosomes.

DArT MARKER	DArT MARKER LINKAGE MAP POSITION (cM)	LINKAGE GROUP/ PSEUDOCHROMOSOME	DArT sequence size (bp)	UNANCHORED SCAFFOLD #	SCAFFOLD SIZE (bp)
ePt-571990	39.98	7	342	24	625,428
ePt-599305	45.43	3	226	50	200,725
ePt-639198	60.54	2	688	118	109,742
ePt-569673	36.45	2	326	134	73,680
ePt-574447	77.42	2	497	134	
ePt-641713	36.62	2	305	134	
ePt-568315	30.79	7	317	207	54,558
ePt-503680	85.13	8	310	207	
ePt-572981	92.18	9	337	460	27,208
ePt-599970	69.7	6	452	491	26,388
ePt-641657	69.7	6	453	491	
ePt-641907	69.7	6	452	491	
ePt-642658	69.7	6	453	491	
ePt-503696	47.35	6	465	499	25,365
ePt-641193	26.65	8	304	556	23,569
ePt-571092	93.5	2	295	578	23,216
ePt-565718	7	6	610	686	22,065
ePt-641578	6.55	6	438	686	
ePt-503229	11.76	10	241	746	18,491
ePt-570410	34.11	9	522	847	16,120
ePt-642696	42.73	9	536	847	
ePt-643222	44.55	11	554	949	14,791
ePt-599337	148.04	1	377	958	16,434
ePt-643196	148.46	1	376	958	
ePt-567002	64.73	8	445	1033	
ePt-640230	44.41	3	292	1165	12,751
ePt-572676	35.65	10	382	1221	12,304
ePt-575708	98.48	8	549	1340	11,269
ePt-644097	98.48	8	558	1340	
ePt-599556	56.67	2	546	1519	10,167
ePt-599364	63.45	8	245	1533	9,961
ePt-570223	30.13	3	399	1585	10,093
ePt-640659	122.09	2	342	1685	9,193
ePt-504097	31.65	8	601	1742	8,856
ePt-565887	51.24	5	580	1843	8,383
ePt-599614	50.38	5	578	1843	
ePt-643170	51.28	5	578	1843	
ePt-570110	81.04	9	229	1891	8,187
ePt-568521	59.84	2	381	1915	8,269
ePt-564907	29.1	9	356	2966	7,743
ePt-573755	34.37	3	383	3233	4,288
ePt-599351	34.43	3	384	3233	
ePt-568568	85.77	5	383	3329	4,170
ePt-572802	47.53	3	263	4064	3,562
ePt-573923	47.51	3	263	4064	
